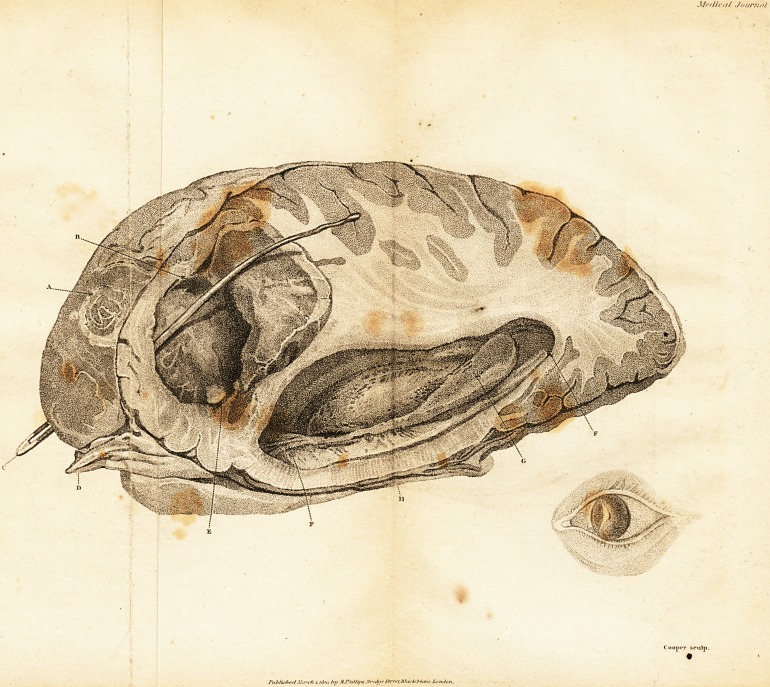# Case of Suppuration of the Brain, Discovered by Dissection, but Not Suspected during Life

**Published:** 1810-02

**Authors:** Henry Earle


Medical and Phyfical Journal.

VOL. XXIII.] FEBRUARY, 1810. [NO. 132.

*Printed for* R. PHILLIPS, *by* W. Thorne, *Red Lion Court, Fleet Street, London.*

### *Case of Suppuration of the Brain, discovered by Dissection, but not suspected during Life.* By JAMES EARLE, Esq.

( With an Engraving. )

MICHAEL KELLY was admitted into St. Bartholomew's Hospital, August the 3d, 1809. His age was about 40, his occupation a mason; he was naturally of a morose irritable disposition, and of a very costive habit of body. At the time of his admission, he had a small sore on his penis, a swelling in his right groin, an ulcer in his throat, and several scabs on his face, head, and over his body; his skin was hot and dry, his tongue furred, and his bowels costive. Prior to his admission, he had used a great deal of mercury in the form of pills, and was evidently labouring under the mercurial fever. He was directed to take some opening medicine, and afterwards salines with antimony. The scabs were fomented, and covered with soft dressings; when they came away, several ulcers were exposed to view with high excavated edges, considerable surrounding inflammation, and were acutely sensible. He continued this plan of treatment for some time; the fever gradually abated, and he improved in health. The only alteration for the first month was, that the sores were three different times fumigated with the sulphurate of mercury, and each time became more irritable and sloughed. During all this time he passed very restless nights, being much troubled in his mind about a promise of marriage which he had made and had broken through; he repeatedly told the nurse that he believed his present affliction was a curse from God, and swore positively that he had never touched but *one* woman who *never* had any venereal complaint. At the end of a month, his health and mouth permitting it, a mercurial course was recommended. He rubbed in a drachm of mercurial ointment for seven nights, when his health became considerably worse; his sores spread and his fever returned; he was now ordered to discontinue his mercury, was removed into a more airy ward, and directed to take salines as before.

Thus far in the history of the case, I am indebted to my friend Mr. C. Wingfield, I myself did not take the patient under my care until the latter end of September; I regret much that it was not in my power to gain any clear information respecting the origin and progress of the disease prior to his admission into the hospital. When I first saw him, his skin was very dry, his tongue covered with a dark brown fur; he had no appetite; his pulse were frequent and wiry; he was very irritable, could not rest at night, and was so debilitated as not to be able to sit up in bed; he had also regular exacerbations of fever at night, and profuse sweats towards morning. The sores were in a very foul state, particularly those on the head and nose: the edges were very hollow; the sloughs of the cellular substance and tendon of the occipito-frontalis, extending in some parts nearly an inch under the skin; the superincumbent skin had a tawny colour, and was very painful, exhibiting altogether one of the most horrible objects I ever beheld. When the scabs and sloughs were removed by bread poultices made with the expressed juice of the carrot, the bone was left bare in several places; it had not the appearance of a carious bone as in the true syphilis, but exactly resembled a bone laid bare by external violence. Light preparations of bark with sulphuric acid were given him every six hours, and Plummer's pill with a grain of opium was directed to be taken night and morning; his bowels were kept regular with the confectio sennæ; his stools at first resembled pitch in consistence and colour, and had no feculent smell. The sores were carefully dressed twice a day with lint dipped in a weak solution of argentum nitratum; the edges were occasionally touched with it in a pure state, with a view to bring them more on a level with the sore, without which it was impossible for them to heal. Under this mode of treatment he improved considerably; the secretions from his bowels were better, and the sores improved; still, however, he rested ill at night. The whole list of narcotics was tried to a great extent, but to very little purpose; they relieved for a short time, but soon lost their effect. The means of relief afterwards adopted were small alterative doses of mercury, as one grain of calomel every second night, and tonics. It was frequently necessary to change his medicine, for he was exceedingly fanciful.

He continued progressively mending until the middle of of November; several of the sores were healed, and others were healing; one, however, on his nose was still irritable, when a small sore on his right cheek became painful and sloughed; immediately almost the sore on the nose improved, a circumstance I have frequently observed to take place. This sore continued to spread till it reached the corner of his mouth, and destroyed the levator anguli oris, in consequence of which the lip hung down, and he was unable to retain his saliva; it was very difficult to keep any dressings on this sore, which made it considerably worse; the discharge dried on the surface of it, forming hard scabs, which kept up continual irritation. I should have mentioned that all the sores, when I first saw him, had this disposition; and I consider that the improvement which took place in them, depended, in a great measure, in the careful removal of these scabs and sloughs twice a day, until the discharge became less in quantity, and more puriform.

On Tuesday, the 21st of November, I placed a small blister on the right temple, thinking that counter irritation might stop the progress of the sore on the cheek, as *it* had done that on the nose.

On the Wednesday night, after having taken a scruple of hyoscyamus and two grains of opium, to procure sleep, he rambled a little, frequently calling upon me; when awake he was perfectly collected. The sore looked a little better the following day, but at night he again was restless, and rambled; the same occurrences took place the two following nights, but in a very slight degree; his appetite had been falling off for some days, and he now refused to take food.

On Sunday, he told me he felt better; the sores *all* looked well, the blister had been kept open; his pulse were now strong and full, about 80, although he could not rise from his pillow. I remarked to Dr. Spalding, who was with me, that I thought it was the last effort of the constitution: the only remarkable thing I observed that day was, that he did not appear to be able to measure distances correctly; he could, however, see me, and answered my questions quite rationally. The following morning, at six o'clock, he terminated his wretched existence.

At five the same evening, I examined his body.—On opening the abdomen, I found the viscera, generally speaking, healthy; the only circumstances worthy of note were, that the gall-bladder was closely contracted round three large gall-stones, about the size and form of large

 dice.

dice. The fæces were tinctured with bile. The spleen was small and pale. The thoracic viscera were also perfectly healthy. I next examined the head, more with a view to see the internal table of the skull, than in any expectation of finding disease. On removing the calvareum, I discovered a considerable slough and thickening of the dura mater, just above the right orbit, opposite to where the bone was laid bare. The internal table did not appear diseased; at one very small place, the bone had been absorbed; it was, however, covered externally with granulations; the external table to some extent was exfoliating kindly. Under this slough I felt a fluctuation. It being very late and dark, I resolved to remove the whole brain, and dissect it at my leisure; with this view, I cut through the dura mater below the slough, and on raising the anterior lobes of the cerebrum, was surprised to find the basis of the cranium between the dura mater and arachnoid coat filled with very fœtid pus. The following morning I carefully laid open the back part of the right lateral ventricle, leaving the anterior part and the abscess untouched; here I found the same fœtid pus, the choroid plexus was devoid of vessels, and had a sphacelated appearance, and the surfaces of the corpus striatum and thalamus were inflamed and ulcerated. At its anterior horn, the ventricle had a large ulcerated opening, with surrounding inflammation, which communicated with the abscess, which was now laid open, and found to occupy near one-third of the substance of the right hemisphere; its sides were covered with white sloughs. The left lateral ventricle was enlarged, its roof being half an inch higher than the corpus callosum; it had probably been filled with pus, the foramen of Monro being very open, but it had escaped by an opening made with the saw into the posterior horn. There was a small opening between the convolutions of the brain into the abscess, just where it rests on the orbital process. Pus was also effused under the tunica arachnoidea in the basis of the brain, which had escaped from the fourth ventricle. The medulla oblongata was rather soft and irregular on its surface.

I have thought the case worthy of being recorded, inasmuch as it shows to what an extent diseased action may go, in the brain, without its even being suspected during life. I merely add the following ideas which I entertained on the subject, as an attempt to explain the phænomena which presented themselves. This state of the brain must have existed for a considerable time; is it not probable then, then, that the diseased action was first set up when the external wounds were in a foul sloughy state? Judging from analogy, this must have been the case; we know that abscesses and sloughs form on the brain from the effects of external violence done to the scalp, and in these cases, the external wounds are in a flabby, unhealthy state; certainly, the state of the sores and the bone for some time prior to death were not sufficient to account for this diseased state of the brain. The means employed, which seemed to have so good an effect on the external wounds, were insufficient to arrest the progress of a disease in a part whose vital powers were so feeble. I may also add, that the disease being once instituted in a circumscribed cavity, the irritating cause must still remain, and increase as the disease increased, or, to use the words of the Poet, "Parva primum mox vires acquiret eundo," and Nature at length finding herself incapable of making reparation, gives up the point, and death closes the scene. With regard to the cause and nature of the disease, it certainly in its appearance and progress was not venereal. It seemed rather to depend on a peculiar state of the constitution, (perhaps dependant on his disturbed state of mind,) which state was much aggravated by the improper use of mercury. I am induced to give this opinion, from having frequently had similar cases, which were relieved by persevering in an alterative and tonic plan of treatment.

## Figures and Tables

**Figure f1:**