# Erratum

**Published:** 1810-03

**Authors:** 


### ERRATUM.

In the first article of our last Number, in the Superscription, for James, *read* Henry Earle.

